# Rapid test for detection of high risk of breast cancer

**DOI:** 10.1186/1897-4287-10-S1-A7

**Published:** 2012-01-12

**Authors:** K Durda, K Jaworska, A Jakubowska, T Dębniak, B Górski

**Affiliations:** 1 International Hereditary Cancer Center, Department of Genetics and Pathology, Pomeranian Medical University, Szczecin, Poland

## 

Direct sequencing of genes is effective method in detection of mutations associated with high predisposition to breast cancer, however is also expensive and time consuming. Therefore, we developed a rapid genetic test for detection of high risk of breast cancer in Polish population. We selected 15 mutations located in four genes associated with high predisposition to breast cancer in Poland: BRCA1 - 5 mutations (Górski et al. IJC 2004) BRCA2 – 5 mutations (Górski et al. IJC 2004, Serrano Fernandez et al. BCRT 2009), CHEK2 – 4 mutations (Cybulski et al. Clin Res. 2006, Cybulski et al. BCRT 2007, Cybulski et al.JMG 2009, Cybulski et al. Clin Genet. 2009, Gronwald et al.BJC 2010) i ATM – 1 mutations (Bogdanowa et al. BCRT 2009).

For genotyping we used the Real-Time PCR technique and custom-made Taqman assays. This test is rapid (results of Taqman genotyping we are available within 2 hours), cheap, easy and sensitive in detection of mutations in genes associated with high predisposition to breast cancer.

In order to meet all sensitivity and quality standards for such tests we are using positive, negative and blinded control in each analysis. The specificity and reproducibility of this test is assessed by repeated analysis of 10% samples. All positive results are confirmed by analysis of second sample from the same patient by independent method eg. PCR-RFLP or sequencing.

